# Evaluation of daily residual pancreatic tumor motion for deep‐inspiration breath‐hold radiotherapy

**DOI:** 10.1002/acm2.70028

**Published:** 2025-02-17

**Authors:** Weihua Mao, Binbin Wu, Kai Ding, Sarah Han‐Oh, Amol Narang

**Affiliations:** ^1^ Department of Radiation Oncology and Molecular Radiation Sciences Johns Hopkins University Baltimore Maryland USA

**Keywords:** CBCT projections, fiducial marker, residual motion

## Abstract

**Purpose:**

Breath‐hold techniques are widely used in radiation therapy to minimize respiratory‐induced tumor or organ‐at‐risk motion. However, residual motion persists, necessitating a reliable daily evaluation method.

**Methods:**

At our institution, fiducial markers serve as surrogates for target localization in pancreatic cancer treatment. We developed an automated method to detect fiducial markers in every projection image of cone‐beam computed tomography (CBCT) scans acquired for patient setup and positioning verification. This method was retrospectively validated using data from nine pancreatic cancer patients.

**Results:**

Residual motion was observed in all patients during breath‐hold maneuvers. Intrafraction target motion in repeated breath‐hold simulation CT scans averaged 1.9 ± 2.2 mm, with a maximum displacement of 8 mm in the superior‐inferior direction. Within a single CBCT scan, residual motion reached up to 7.3 mm, with an average drifting range of 3.8 ± 1.1 mm across 94 CBCT scans. The average standard deviation of drift was 1.5 ± 0.5 mm. Significant drift (1.3 ± 1.2 mm) and inter‐breath‐hold gaps (2.6 ± 2.0 mm) were detected within the same CBCT scan.

**Conclusion:**

Our method enables daily residual motion assessment without additional equipment or extra radiation exposure. This information is critical for refining planning margins in online adaptive radiation therapy, improving treatment precision and patient safety.

## 1 INTRODUCTION

A key factor in pancreatic cancer radiation therapy is the proper positioning of the patient and the management of respiratory motion to ensure the accurate delivery of prescription doses to targets while minimizing toxicity to surrounding critical organs.^1^ A large planning margin had to be added to expand the treatment volume to ensure target coverage; however, this approach could increase the risk of duodenum toxicity.^2^ Deep inspiration breath‐hold (DIBH) has been used to reduce respiratory motion during treatment simulation, cone‐beam computed tomography (CBCT) acquisition for patient positioning, verification, and treatment delivery.^3^, ^4^, ^5^ However, residual motion during breath‐hold (BH) has been reported by several groups. Using an active breathing coordinator (ABC, Elekta, Stockholm, Sweden) BH, Dawson et al. found large intrafraction excursion of implanted hepatic microcoils.^6^ Repeat CT scans with ABC DIBH were studied by multiple groups to estimate residual motion.^7^, ^8^ Blessing et al. manually determined diaphragm dome positions on CBCT projections to assess residual motion with ABC DIBH.^9^ Ultrasound was used to detect diaphragmatic dome positions, comparing with those manually determined in CBCT projections.^10^, ^11^ Zeng et al. studied intermittent kilovoltage images to detect markers with manual initialization.^3^ It is essential to develop an automatic procedure for assessing these motions daily for each patient. At our institution, radio‐opaque fiducial markers are placed endoscopically before radiation therapy and serve as surrogates for targets. Since each CBCT scan lasts about 40 s, the fiducial positions on CBCT projections record daily residual target motion. We present a fully automatic method to evaluate daily fiducial residual motion based on CBCT projections.

## 2 METHODS

### 2.1 Patient data and pre‐treatment motion

Nine pancreatic cancer patients treated since December 2023 were enrolled in this retrospective study under an institutional review board protocol (IRB00395200). Two to three fiducial markers were implanted endoscopically in the pancreas of each patient. These LumicCoil STRIGHT platinum fiducial markers (Boston Scientific, Marlborough, Massachusetts, USA) had a diameter of 0.48 mm and a length of 5 mm. Patients were immobilized using a Vac‐Lok (CIVCO Medical Solutions, Coralville, Iowa, USA) with a Wing Board (CIVCO Medical Solutions, Coralville, Iowa, USA). An active breathing coordinator (ABC, Elekta, Stockholm, Sweden) was employed for DIBH. Patients were coached to breathe in, breath out, and hold their breath. Per institution protocol,^4^, ^5^, ^12^ four computed tomography (CT) simulation scans were acquired after contrast agent injection during separate DIBH sessions using the Brilliance Big Bore CT simulators (Philips Medical Systems, Cleveland, Ohio, USA). One set of CT images was selected for contouring and treatment planning in RayStation (RaySearch Laboratories AB, Stockholm, Sweden). The slice thickness of the CT images was 2 mm, while planar pixel size was approximately 1.4 mm. Fiducials were contoured in the bone window ([450, 1600] HU). Volumetric modulated arc therapy (VMAT) with two arcs was planned. Either stereotactic body radiation therapy (33 or 40 Gy in five fractions) or conventionally fractionated radiation therapy (54–63 Gy in 25–30 fractions) was prescribed. Detailed patient demographics and prescription information are listed in Table 1. Patients were treated using two VersaHD (Elekta, Stockholm, Sweden) linear accelerators. Partial arc (200‐degree) rotation CBCT was used with settings of 120 kV, 40 mA, and 40 ms. The source‐to‐axis distance (SAD) was 1000 mm, the source‐to‐imager distance (SID) was 1536 mm, the projection image resolution was 512 × 512, and the pixel size was 0.8 mm. The maximum BH duration was 25 s for each patient. Each CBCT scan was acquired in two consecutive BHs. Reconstructed CBCT images were rigidly registered to the planning CT based on bony structures and finalized using fiducial markers. After multiple reviews, six‐dimensional corrections were sent to the Hexapod (Elekta, Stockholm, Sweden) to adjust the treatment couch. Verification CBCT scans were acquired immediately before and/or during the middle of treatment. Treatment beams were delivered under identical DIBH conditions.

**TABLE 1:** List of patient information, average fiducial marker motions among four CT sets, and contouring expansions from gross tumor volume (GTV) to clinical target volume (CTV) and from CTV to planning treatment volume (PTV).

			Prescription		Average fiducial marker motions in CTs (mm)		Contour expansion (mm)	
Patient ID	Gender	Age	Dose (Gy)	# of Fx	SI	3D	GTV → CTV	CTV → PTV
#1	M	65	40.00	5	2.0	3.0	3	2
#2	F	57	33.00	5	4.0	4.1	4 / 2	2
#3	M	67	40.00	5	3.3	4.6	5	3
#4	F	76	33.00	5	1.3	1.7	3	2
#5	M	60	40.00	5	0.0	0.7	3	2
#6	M	81	33.00	5	1.0	1.9	3	2
#7	M	59	58.80	28	1.3	2.8	4	5
#8	M	46	54.00	30	3.3	4.9	3	7
#9	F	76	63.00	28	1.0	2.2	4	5

Planning CT images and structures, CBCT projection data, and ABC results were exported and de‐identified from clinical systems. All analyses were performed using in‐house MATLAB programs (MathWorks, Natick, Massachusetts, USA). Fiducial mass centers were calculated within fiducial structures to determine fiducial positions in the planning CT. Individual and average fiducial positions were compared across four sets of simulation CT images by registering the planning CT to the other three repeated CT sets, focusing on fiducials. The distances between fiducial markers across the four sets of CT images were calculated.

### 2.2 Marker detection

**FIGURE 1:**
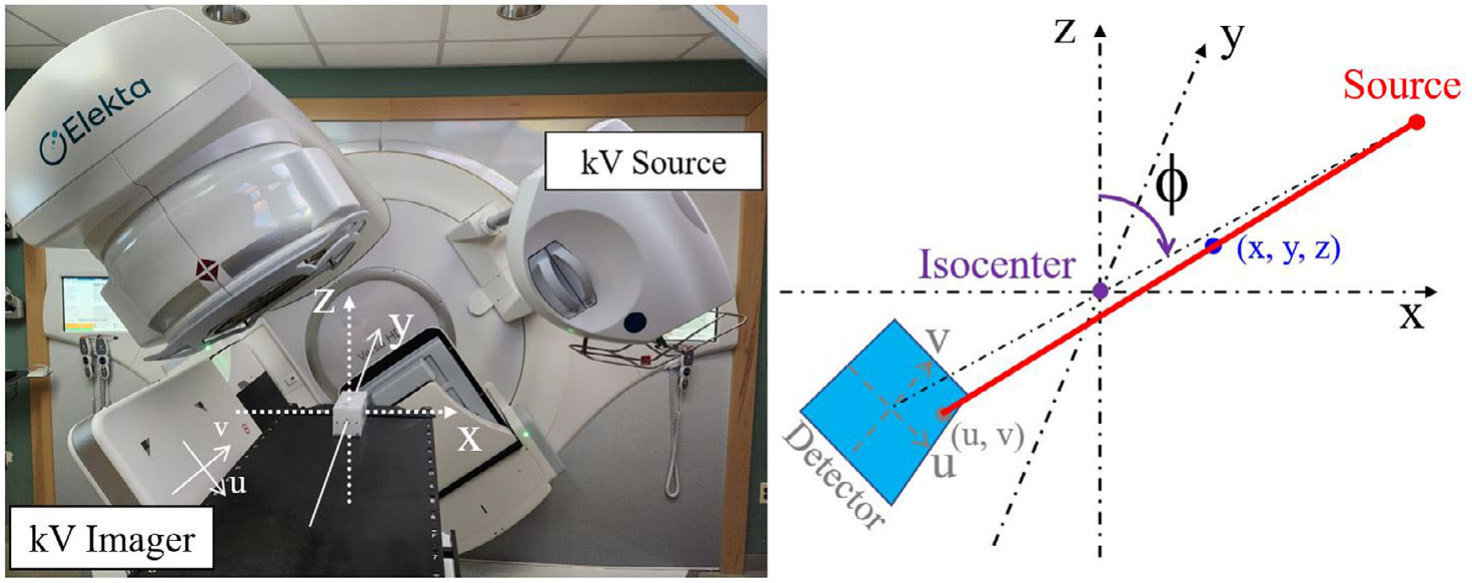
Schematic diagram of the coordinate systems.

Figure 1 illustrates both the linac and imaging coordinate systems (IEC 61217 Standard). The origin is set at the isocenter. The X, Y, and Z axis directions represent the horizontal (lateral), longitudinal (inferior‐superior), and vertical (posterior‐anterior) directions, respectively. Projection images are in a two‐dimensional (2D) coordinate system (u, v), with the origin at the center of the imager, which rotates with the gantry. Equations (1) and (2) predict the projected location (u, v) of a fiducial at (x, y, z).^13^

(1) $$u=\frac{\mathrm{SID}\cdot \left(x\cdot \cos\phi -z\cdot \sin\phi \right)}{\mathrm{SAD}-x\cdot \sin\phi -z\cdot \cos\phi }$$

(2) $$v=\frac{\mathrm{SID}\cdot y}{\mathrm{SAD}-x\cdot \sin\phi -z\cdot \cos\phi }$$

**FIGURE 2:**
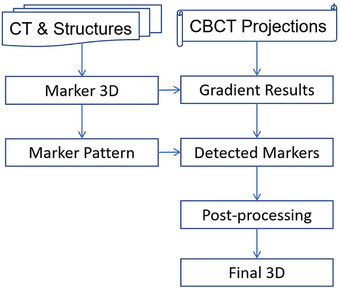
Flow chart of marker detection.

**FIGURE 3:**
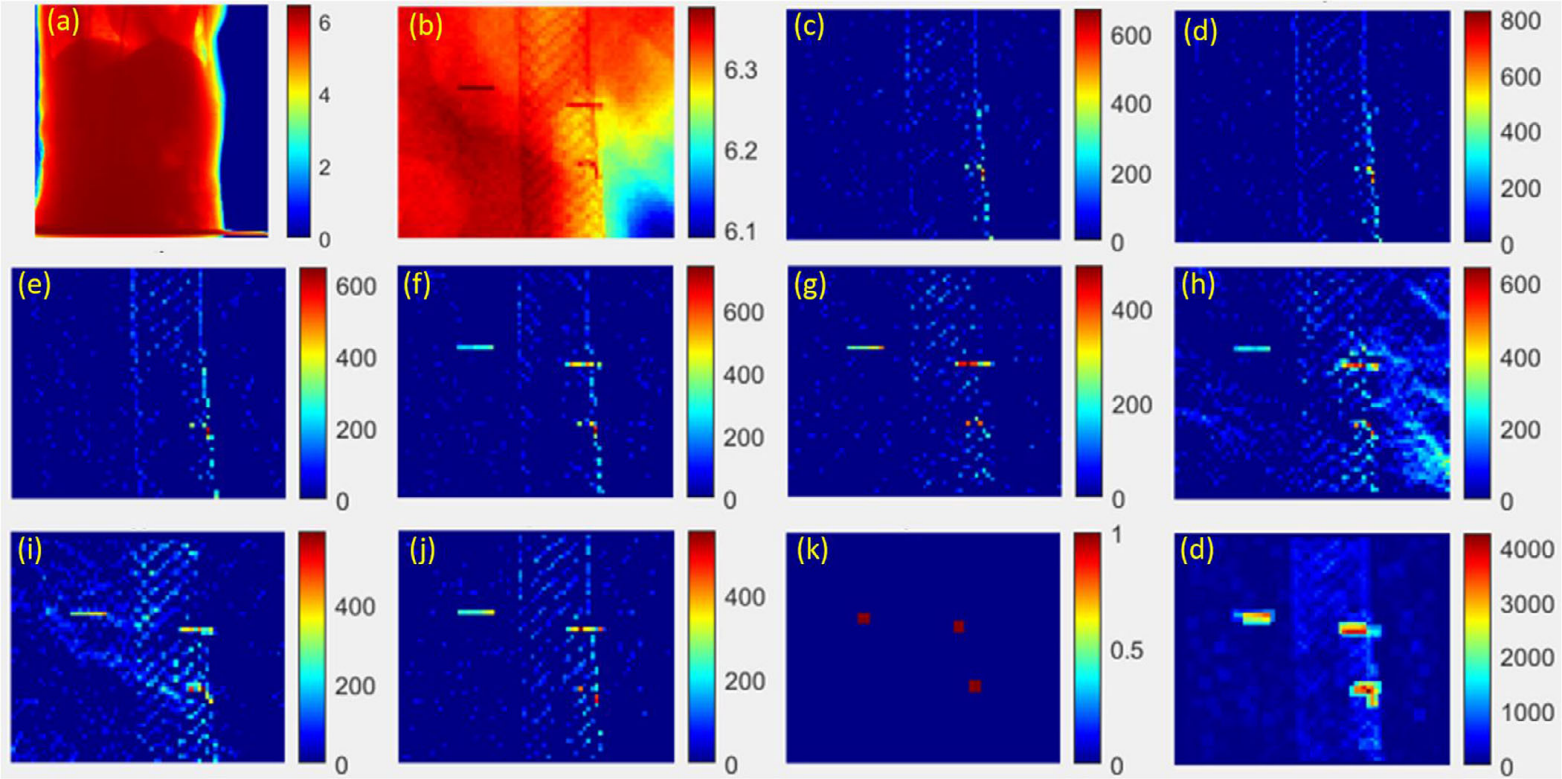
Imaging processing. A sample projection acquired at 252⁰. (a) Full projection image; (b) image patch; (c) single‐pixel gradient in u direction (Gu); (d) left double‐pixel gradient in u direction (Guu1); (e) right double‐pixel gradient in u direction (Guu2); (f) oblique gradient in uv direction (Guv); (g) single‐pixel gradient in v direction (Gv); (h) upper double‐pixel gradient in v direction (Gvv1); (i) lower double‐pixel gradient in u direction (Gvv2); (j) oblique gradient in vu direction (Gvu); (k) expected positions; (l) total gradient and match results.

Figure 2 illustrates the flow chart of the marker detection algorithm. Projection image analyses focused on regions around fiducial projection locations estimated using Equations (1) and (2) for each projection angle, as shown in Figure 3a. A patch of the image was selected based on initial expected marker positions (Figure 3b). Simple intensity differences as gradients or ridge heights, were calculated in four directions (horizontal, vertical, and two oblique directions). Since the thickness of the fiducial projection is only one or two pixels, gradients from both sides of within a width of one or two pixels were calculated for each pixel *I*(nu, nv) following Equations (3)–(13), while nu and nv represented the pixel locations in the u‐ and v‐directions, respectively.

(3) $$gu1\;\left(nu,nv\right)=I\left(nu,nv\right)-I\left(nu-1,nv\right),$$

(4) $$gu2\;\left(nu,nv\right)=I\left(nu,nv\right)-I\left(nu+1,nv\right),$$

(5) $$guu1\;\left(nu,nv\right)=I\left(nu,nv\right)-I\left(nu-2,nv\right),$$

(6) $$guu2\;\left(nu,nv\right)=I\left(nu,nv\right)-I\left(nu+2,nv\right),$$

(7) $$gv1\;\left(nu,nv\right)=I\left(nu,nv\right)-I\left(nu,nv-1\right),$$

(8) $$gv2\;\left(nu,nv\right)=I\left(nu,nv\right)-I\left(nu,nv+1\right),$$

(9) $$gvv1\;\left(nu,nv\right)=I\left(nu,nv\right)-I\left(nu,nv-2\right),$$

(10) $$gvv2\;\left(nu,nv\right)=I\left(nu,nv\right)-I\left(nu,nv+2\right),$$

(11) $$guv1\;\left(nu,nv\right)=I\left(nu,nv\right)-I\left(nu-1,nv-1\right),$$

(12) $$guv2\;\left(nu,nv\right)=I\left(nu,nv\right)-I\left(nu+1,nv+1\right),$$

(13) $$gvu1\;\left(nu,nv\right)=I\left(nu,nv\right)-I\left(nu-1,nv+1\right),$$

(14) $$gvu2\;\left(nu,nv\right)=I\left(nu,nv\right)-I\left(nu+1,nv-1\right).$$

All gradients were screened using a preset threshold, and any gradient result below the threshold was reset to zero. The square roots of their products were used as heights in each direction (Figures 3c–j) using Equations (15)–(22):

Single pixel gradient in u direction (Figure 3c):

(15) $$Gu\left(nu,nv\right)=\sqrt{gu1\left(nu,nv\right)\cdot gu2\left(nu,nv\right)}\;$$

Left double‐pixel gradient in u direction (Figure 3d):

(16) $$Guu1\left(nu,nv\right)=\sqrt{gu1\left(nu,nv\right)\cdot guu2\left(nu,nv\right)}\;$$

Right double‐pixel gradient in u direction (Figure 3e):

(17) $$Guu2\left(nu,nv\right)=\sqrt{gu2\left(nu,nv\right)\cdot guu1\left(nu,nv\right)}\;$$

Oblique gradient in uv direction (Figure 3f):

(18) $$Guv\left(nu,nv\right)=\sqrt{guv1\left(nu,nv\right)\cdot guv2\left(nu,nv\right)}\;$$

Single‐pixel gradient in v direction (Figure 3g):

(19) $$Gv\left(nu,nv\right)=\sqrt{gv1\left(nu,nv\right)\cdot gv2\left(nu,nv\right)}\;$$

Upper double‐pixel gradient in v direction (Figure 3h):

(20) $$Gvv1\left(nu,nv\right)=\sqrt{gv1\left(nu,nv\right)\cdot gvv2\left(nu,nv\right)}\;$$

Lower double‐pixel gradient in v direction (Figure 3i):

(21) $$Gvv2\left(nu,nv\right)=\sqrt{gv2\left(nu,nv\right)\cdot gvv1\left(nu,nv\right)}\;$$

Oblique gradient in vu direction (Figure 3j):

(22) $$Gvu\left(nu,nv\right)=\sqrt{gvu1\left(nu,nv\right)\cdot gvu2\left(nu,nv\right)}\;$$

The relative positions of all fiducials were calculated using 3D positions of fiducials obtained in sim CT images based on Equations (1) and (2) and used as a pattern (Figure 3k) to match and locate the fiducials. The summation of the above eight gradients from Equations (15)–(22) matched and highlighted in Figure 3l.

### 2.3 Post‐analysis

A screening analysis was performed on 2D fiducial positions to remove outliers based on expected position trendlines. For series position of marker position, $u_{i}$, at projection angle, $\phi_{i}$, three parameters were calculated using Equations (23)–(25):

(23) $$a_{i}=SID\cdot \cos\phi_{i}+u_{i}\cdot \sin\phi_{i}$$

(24) $$b_{i}=u_{i}\cdot \cos\phi_{i}-SID\cdot \sin\phi_{i}$$

(25) $$c_{i}=SAD$$

**FIGURE 4:**
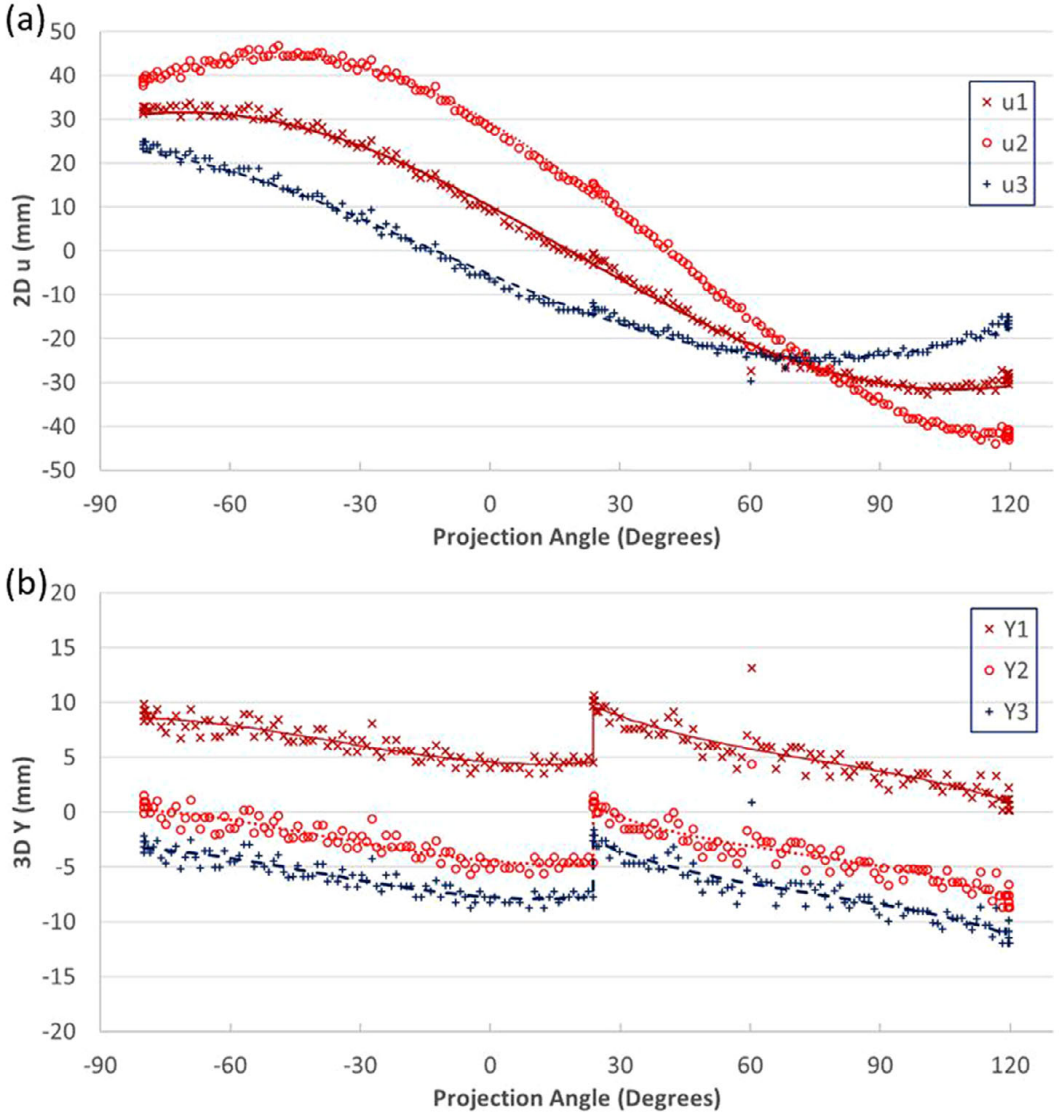
Screening process. (a) 2D u results (symbols) and expected locations (lines); (b) 3D Y results (symbols) and expected locations (lines).

Lateral (X) and vertical (Z) coordinates of each fiducial marker were calculated based on a series of lateral projection positions (u) from the entire scan, as described in Equations (26) and (27):

(26) $$X=\frac{\left(\sum_{i=1}^{N}a_{i}\cdot c_{i}\right)\left(\sum_{i=1}^{N}b_{i}\cdot b_{i}\right)-\left(\sum_{i=1}^{N}b_{i}\cdot c_{i}\right)\left(\sum_{i=1}^{N}a_{i}\cdot b_{i}\right)}{\left(\sum_{i=1}^{N}a_{i}\cdot a_{i}\right)\left(\sum_{i=1}^{N}b_{i}\cdot b_{i}\right)-\left(\sum_{i=1}^{N}a_{i}\cdot b_{i}\right)\left(\sum_{i=1}^{N}a_{i}\cdot b_{i}\right)}$$

(27) $$Z=\frac{\left(\sum_{i=1}^{N}a_{i}\cdot a_{i}\right)\left(\sum_{i=1}^{N}b_{i}\cdot c_{i}\right)-\left(\sum_{i=1}^{N}a_{i}\cdot b_{i}\right)\left(\sum_{i=1}^{N}a_{i}\cdot c_{i}\right)}{\left(\sum_{i=1}^{N}a_{i}\cdot a_{i}\right)\left(\sum_{i=1}^{N}b_{i}\cdot b_{i}\right)-\left(\sum_{i=1}^{N}a_{i}\cdot b_{i}\right)\left(\sum_{i=1}^{N}a_{i}\cdot b_{i}\right)}$$

(28) $$y_{i}=\frac{v_{i}}{\mathrm{SID}}\left(SAD-X\cdot \sin\phi -Z\cdot \cos\phi \right)$$

where *N* is the number of projections used for this series. These coordinates were then used to calculate expected projections for each scan using Equations (1) and (2) (as shown in Figure 4a). Any projection with a detected lateral position deviating from expected detected positions by more than 5 mm was regarded as an outlier and excluded from further analysis. The 3D longitudinal (Y) coordinates of each fiducial marker were calculated for every projection using Equation (28) with calculated X and Z positions. Screening analyses were performed for each BH separately. A third‐order polynomial curve fitting was applied to the longitudinal coordinates. Any projections with detected longitudinal positions deviating from the curve‐fitting results by more than 3 mm were regarded as outliers and excluded (Figure 4b).

The 3D longitudinal coordinates were analyzed for each BH and for the entire scan (both BHs). Average positions, maximum deviation from the average, and standard deviations were calculated. The difference in average positions between the two BHs was calculated for each scan. A gap, representing the position difference between the end of the first BH and the beginning of the second BH, was calculated for each scan.

## 3 RESULTS

### 3.1 Pre‐treatment motion

It was found that the fiducials could be misaligned by up to three slices (6 mm) across four sets of CT images acquired under DIBH. Table 1 lists patient information and prescription schemes based on a review of charts and the treatment planning system. The average (Avg) superior‐inferior (SI) motions of fiducial markers were compared across the four sets of CT images as shown in Table 1, along with contouring expansion from gross tumor volume (GTV) to clinical target volume (CTV) and from CTV to planning treatment volume (PTV).

### 3.2 Daily motion

**FIGURE 5:**
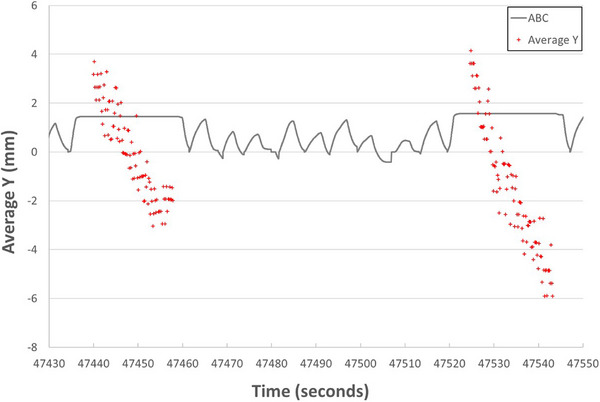
Comparison of average fiducial longitudinal locations (Y) and ABC results.

**FIGURE 6:**
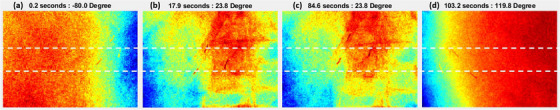
Comparison of fiducial locations at beginning and ending of each breath‐hold for a scan.

CBCT projections from 6 to 16 acquisitions per patient were successfully analyzed. In total, 18 649 frames of 2D projection images were acquired, and fiducials were successfully detected on 17 402 frames of images. Residual motions in the longitudinal direction were observed. Figure 5 illustrates the average longitudinal positions of fiducials during two BHs in a CBCT scan. Actual fiducial locations are compared in Figure 6 for reference. Significant drift was observed during the same BH. Four key positions were defined as the start and end of each BH. The drifts in the longitudinal direction were calculated for each scan. The drifting range in each BH and between two BHs was also calculated. Statistical results for each patient are listed in Table 2. The average drifting range from mean position during a scan was 3.8 ± 1.1 mm, with a maximum range of 7.3 mm. The average standard deviation of drifting was 1.5 ± 0.5 mm. The difference in average positions between two BHs per scan was 1.3 ± 1.2 mm, while the maximum drift was up to 5.1 mm. The gaps between two BHs are 2.6 ± 2.0 mm, with a maximum gap of 8.2 mm.

**TABLE 2:** Results of average fiducial longitudinal positions from 2D projections. Average (Avg) and standard deviation (std) are calculated for Maximum Deviation (Max Dev) and Standard Deviation (Std Dev) of average fiducial longitudinal positions in every Breath‐Hold or both Breath‐Holds.

		1st breath‐hold				2nd breath‐hold				Both breath‐holds							
		Max Dev		Std Dev		Max Dev		Std Dev		Max Dev		Std Dev		Avg difference between breath‐holds		Gap between breath‐holds	
Patient ID	Num Of scans	Avg	Std	Avg	Std	Avg	Std	Avg	Std	Avg	Std	Avg	Std	Avg	Std	Avg	Std
1	8	2.2	0.5	0.8	0.3	2.2	0.7	1.1	0.4	3.6	1.3	1.6	0.8	2.2	2.0	2.7	2.0
2	15	3.2	0.6	1.4	0.2	2.2	1.3	0.8	0.6	3.5	0.8	1.5	0.4	1.2	1.1	3.0	2.0
3	6	3.0	0.6	1.0	0.3	3.5	0.9	1.0	0.3	4.1	1.2	1.3	0.5	1.2	1.2	2.9	2.1
4	9	3.8	0.9	1.5	0.1	4.0	1.0	1.8	0.5	4.6	1.1	1.8	0.4	1.4	0.9	2.9	1.2
5	9	2.0	0.9	0.8	0.4	1.9	0.7	0.7	0.3	2.6	0.4	1.0	0.3	1.0	0.9	1.8	1.1
6	13	3.1	0.8	1.2	0.4	4.3	1.8	1.6	0.6	4.9	1.4	1.7	0.5	1.3	1.1	4.2	2.4
7	16	3.3	0.7	1.2	0.2	3.0	0.6	1.1	0.2	3.8	0.5	1.2	0.1	0.7	0.5	1.4	1.2
8	10	3.6	1.0	1.7	0.8	1.7	1.1	0.7	0.4	4.2	1.0	2.0	0.7	2.1	1.8	3.3	2.3
9	8	2.6	0.8	0.9	0.2	1.5	0.6	0.6	0.2	2.9	0.6	1.0	0.5	1.1	1.3	1.7	1.8

Average (Avg) and standard deviation (Std) are calculated for average differences between breath‐holds and the gap between end of the 1st breath‐hold and beginning of the 2nd breath‐hold.

## 4 DISCUSSION

It should be noted that all simulation CT and CBCT projections were acquired under fixed DIBH conditions for every patient. Ideally, fiducial marker locations should remain in the same positions. However, residual motions were first observed during multiple simulation CT scans. Additional contour margins (expansion from GTV to CTV) were added to accommodate these residual motions, as listed in Table 1. CBCT volumetric image reconstruction averages out residual motions and reconstructs static 3D images. Our method is capable of extracting daily residual motions from CBCT projections. Although only motions lasting about 40 s were obtained, they reveal daily motion variations that provide significantly more information than repeated simulation CT scans.

An appropriate planning margin should be applied to account for residual motions. Our current margin is 3 mm or larger to expand the GTV to the CTV, accounting for DIBH motion uncertainties. The standard deviation of fiducial marker positions was 1.5 ± 0.5 mm. It should be noted that the imager resolution is 0.8 mm, corresponding to 0.5 mm at the isocenter. Roughly, the margin is twice the standard deviation, meaning the margin covers approximately 95% of marker positions and should be clinically reasonable to ensure accurate dose delivery to targets. However, the standard deviation of motion ranged from 0.6 to 3.4 mm across 94 scans. As listed in Table 2, the patient's standard deviation of motion standard deviation indicates the daily variations, which might be up to 0.8 mm. This margin should be patient‐specific and verified daily.

Unlike the algorithms reported in the literature,^3^, ^14^ this method is a fully automatic procedure that requires nv dqo manual labeling. This method offers a cost‐free daily assessment of the residual motion during breath‐holding since it does not require any additional equipment or procedures and does not deliver any extra radiation doses to patients. If plans with different planning margins are prepared in advance, the plan with the appropriate margin will be selected based on the daily motion pattern, functioning as a simplified adaptive radiation therapy.

One drawback of this method is that motion in the directions orthogonal to the longitudinal direction could not be continuously tracked, as the u‐direction keeps changing during gantry rotation. Fortunately, the largest motion due to respiration typically occurs in the longitudinal (Y) direction, which can be continuously tracked.

This method uses marker distributions in simulation CT images to match markers in projections. However, the markers’ relative positions may change after the simulation CT acquisition, which can make it difficult to distinguish each marker correctly. Further studies are ongoing.

This is a preliminary study, with only nine patients enrolled. It is too early to draw meaningful conclusions about predicting daily motion range based on the results of simulation CT or other information. Further studies will be conducted with more patients enrolled in the near future. This method evaluated motion only during CBCT acquisition, which may differ from the motion occurring during the subsequent beam‐on treatment. It is necessary to validate whether the residual motion pattern remains consistent throughout a treatment fraction. With more data collected, future studies will focus on analyzing day‐to‐day changes in residual motion patterns.

In addition, future studies will aim to correlate marker motion with other motions, such as diaphragm and skin motion, which can potentially be monitored in real‐time. Results from other technologies, including ultrasound,^10^, ^11^ cine imaging from MR‐guided radiation therapy,^15^, ^16^ and continuous tracking using Calypso systems,^17^, ^18^ or particularly ExacTrac‐based internal marker and surface tracking,^19^ could be compared in internal and external motion correlation studies. Such comparisons would be a significant enhancement to CBCT projection image‐based tracking, as this technique is readily available on most modern linear accelerators.

## 5 CONCLUSION

Fiducial residual motions under BH could be automatically assessed daily before radiation therapy without any additional cost.

## AUTHOR CONTRIBUTIONS

Dr Weihua Mao led the project, overseeing data collection, algorithms development, data analysis, and manuscript preparation. Drs. Binbin Wu, Kai Ding, Sarah Han‐Oh, and Amol Narang contributed to the project by assisting with data collection, participating in data analysis, and contributing to the preparation of the manuscript.

## CONFLICT OF INTEREST STATEMENT

The authors declare no conflicts of interest.
